# Assessment of warranty time for dobutamine stress magnetic resonance imaging in 3138 consecutive patients: a bi-center study

**DOI:** 10.1186/1532-429X-14-S1-O14

**Published:** 2012-02-01

**Authors:** Sebastian Kelle, Eike Nagel, Amedeo Chiribiri, Rolf Gebker, Christopher Schneeweis, Alexander Berger, Evangelos Giannitsis, Eckart Fleck, Hugo A Katus, Grigorios Korosoglou

**Affiliations:** 1 Cardiology, German Heart Institute Berlin, Berlin, Germany; 2 Division of Imaging Sciences, Kings College London, London, UK; 3 Cardiology, University of Heidelberg, Heidelberg, Germany

## Summary

We aimed to determine the prognostic value of wall motion assessment during high-dose dobutamine stress cardiac magnetic resonance imaging (DCMR) for the prediction of hard cardiac events in a large patient cohort and within a long follow-up duration. DCMR can accurately identify between patients with inducible ischemia who are at increased risk for future hard cardiac events and revascularization procedures. Within the ‘primary warranty time’ of 3 years after DCMR excellent outcomes (annual hard cardiac event rate <1%) are recorded in patients with normal findings.

## Background

We aimed to determine the prognostic value of wall motion assessment during high-dose dobutamine stress cardiac magnetic resonance imaging (DCMR) for the prediction of hard cardiac events in a large patient cohort and within a long follow-up duration.

## Methods

Consecutive patients with suspected or known coronary artery disease underwent DCMR at two experienced cardiac-MRI centers (German Heart Institute Berlin and Heidelberg University Hospital), using a standard protocol with a 1.5T Philips MR-scanner. Wall motion was assessed at rest and during maximum stress, and outcome data including cardiac death and non-fatal myocardial infarction (defined as hard cardiac events) and ‘late’ revascularization (percutaneous coronary intervention or coronary artery bypass grafting) performed >90 days after the MR-scans were prospectively collected at least 6 months after DCMR.

## Results

**Figure 1:**
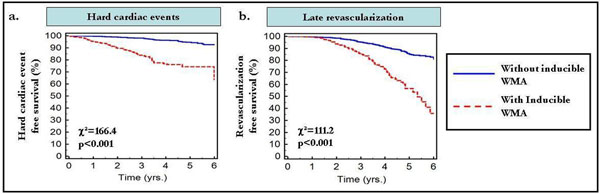


Follow-up data were available in 3138 consecutive patients (mean follow-up 3.3±1.7 years, range 0.5 and 9.7 years). Hard cardiac events occurred in 183 (5.8%) patients during the follow-up period. Patients with an inducible wall motion abnormality (WMA) experienced hard cardiac events and late revascularization at significantly higher rates (hazard ratio (HR): 6.9; 95%; confidence interval (CI): 4.9-9.7, and HR=3.8; 95%CI=2.9-5.0, respectively, p<0.001 for both) (Figure 1) compared to those with normal findings. Within the first 3 years of follow-up excellent outcomes were recorded for patients with normal wall motion during stress in regard to hard cardiac events and late revascularization procedures (annual hard cardiac event rate of 0.6% and revascularization rate of 1.6%). Over the following 3 years of follow up, annual event rates rose for both hard events and revascularization to 1.6% and 3.2%, respectively.

Using multivariate regression analysis, inducible WMA during stress, yielded the strongest independent prognostic value for both, hard cardiac events and late revascularization, clearly surpassing that of clinical and baseline MR-parameters (HR=7.4, 95%CI=3.3-16.7 for hard cardiac events and HR=3.1, 95%CI=1.7-5.5 for late revascularization, p<0.001).

**Figure 2:**
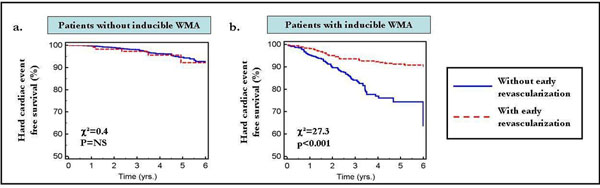


Interestingly, patients without inducible ischemia who underwent early revascularization yielded similar hard cardiac event rates, compared to those who were continued on medical treatment. Conversely, patients with inducible ischemia and early revascularization yielded significantly improved prognosis compared to patients with inducible ischemia who were continued on medical treatment (Figure 2).

## Conclusions

DCMR can accurately identify between patients with inducible ischemia who are at increased risk for future hard cardiac events and revascularization procedures. Within the ‘primary warranty time’ of 3 years after DCMR excellent outcomes (annual hard cardiac event rate <1%) are recorded in patients with normal findings.

## Funding

None.

